# Advanced Molecular Dynamics Approaches to Model a Tertiary Complex APRIL/TACI with Long Glycosaminoglycans

**DOI:** 10.3390/biom11091349

**Published:** 2021-09-12

**Authors:** Mateusz Marcisz, Martyna Maszota-Zieleniak, Bertrand Huard, Sergey A. Samsonov

**Affiliations:** 1 Faculty of Chemistry, University of Gdańsk, ul. Wita Stwosza 63, 80-308 Gdańsk, Poland; mateusz.marciszm@gmail.com (M.M.); m.maszota.zieleniak@gmail.com (M.M.-Z.); 2 Intercollegiate Faculty of Biotechnology of UG and MUG, ul. Abrahama 58, 80-307 Gdańsk, Poland; 3 Laboratory TIMC-IMAG, University Grenoble-Alpes, CNRS UMR 5525, 38700 La Tronche, France; bertrand.huard@univ-grenoble-alpes.fr

**Keywords:** van der Waals replica exchange molecular dynamics, long glycosaminoglycans, APRIL, APRIL receptors, MM/GBSA

## Abstract

Glycosaminoglycans (GAGs) are linear anionic periodic polysaccharides participating in a number of biologically relevant processes in the extracellular matrix via interactions with their protein targets. Due to their periodicity, conformational flexibility, pseudo-symmetry of the sulfation pattern, and the key role of electrostatics, these molecules are challenging for both experimental and theoretical approaches. In particular, conventional molecular docking applied for GAGs longer than 10-mer experiences severe difficulties. In this work, for the first time, 24- and 48-meric GAGs were docked using all-atomic repulsive-scaling Hamiltonian replica exchange molecular dynamics (RS-REMD), a novel methodology based on replicas with van der Waals radii of interacting molecules being scaled. This approach performed well for proteins complexed with oligomeric GAGs and is independent of their length, which distinguishes it from other molecular docking approaches. We built a model of long GAGs in complex with a proliferation-inducing ligand (APRIL) prebound to its receptors, the B cell maturation antigen and the transmembrane activator and calcium modulator and cyclophilin ligand interactor (TACI). Furthermore, the prediction power of the RS-REMD for this tertiary complex was evaluated. We conclude that the TACI–GAG interaction could be potentially amplified by TACI’s binding to APRIL. RS-REMD outperformed Autodock3, the docking program previously proven the best for short GAGs.

## 1. Introduction

Despite the recent advances in molecular docking of glycosaminoglycan (GAG) oligosaccharides, it still remains a challenge to dock longer GAGs [1]. The main reason for this is the physical–chemical nature of GAGs. They are long, periodic, and linear polysaccharides. They are negatively charged and manifest different binding and conformational properties based on their sulfation pattern and negative charge distribution [2]. GAGs are built of disaccharide units consisting of amino sugars and uronic acid or galactose [3]. Depending on their arrangement and sulfation pattern, those units may display 408 [4] variants, of which 202 are found in mammals [5,6]. Although GAG’s certain binding specificity has been observed in several biologically relevant systems [7,8,9], protein–GAG interactions are often predominantly electrostatics-driven, and their binding energies correlate with the GAG net charge [10,11,12,13]. Despite the fact that computational studies of GAGs persist as a general challenge due to the required conformational sampling and their periodicity, there are numerous successful studies on proteins complexes with shorter GAG oligosaccharides (of length up to octasaccharides) [1,14,15,16,17,18]. On the other hand, there are very few studies that focus on longer GAG molecules in complexes with proteins. The reason for that is that there is no appropriate tool for the docking long GAGs to properly account for their conformational space and periodicity. In the case of Autodock3, which has been shown the most accurate tool for docking GAGs [19], there is a limitation of 32 torsional degrees of freedom for the docked molecule, which makes a docking for bigger GAG molecules practically rigid. Dynamic molecular docking allows for flexible docking of GAGs of arbitrary length, implementing targeted MD toward the *a priori* known binding region on the protein surface [20]. However, in case there are several potential binding regions or a binding region is too extensive and/or the ligand is particularly long, this approach is computationally too expensive. The next approach designed to overcome this issue of GAG length is a fragment-based method, in which trimeric GAGs are docked with Autodock3 and further assembled into longer chains [21]. This method also has a potential flaw since it may fail to properly dock when the GAG binding domain has some negatively charged residues that restrict the search of favorable binding patches; thus, short GAG probes cannot be docked near those residues, rendering the length of the assembled long GAG chain limited. The RS-REMD (replica exchange molecular dynamics with repulsive scaling) method [22,23] is not restricted by any of the above-mentioned limits. Moreover, it has been proven that this method is appropriate to dock GAGs [24]. In RS-REMD, effective pairwise radii are increased in different Hamiltonian replicas. In GAG–protein complexes, very often, electrostatic interactions play a main role by establishing strong charge–charge interactions and, therefore, limiting dissociation or any dramatic conformational changes allowing for avoiding binding in a local minimum. Increasing pairwise van der Waals radii as it is done in RS-REMD (while not affecting other types of interactions for the system) can be helpful to overcome this challenge. In this way, the mentioned method allows for a robust and extensive search for the proper binding poses on the complete protein surface, allowing, at the same time, for full flexibility of the docked molecule and the receptor side chains. In this study, a 24-mer and a 48-mer of heparin (HP) were docked to two complexes of a proliferation-inducing ligand (APRIL) protein and its receptors—the transmembrane activator and calcium modulator and cyclophilin ligand interactor (TACI) and the B cell maturation antigen (BCMA). APRIL is a member of the TNF superfamily [25] that was shown to bind GAGs (chondroitin sulfate and heparan sulfate) [13,26,27,28]. Such binding is thought to mediate APRIL’s oligomerization and, therefore, enable its role in cell signaling [29]. The GAG binding region on APRIL’s surface is located near the N-terminus of the protein alongside a stretch of positively charged lysine residues [13]. Additionally, it was reported that the C-terminus spatially close to the N-terminus, together with several arginine residues on the side of the protein, also contribute to GAG binding [13]. While this binding of GAGs to APRIL is believed to be a facilitation agent for its binding to receptors, BCMA and TACI [30,31], it has been shown that BCMA does not bind to HSPG (heparan sulfate proteoglycan) [32,33]. On the other hand, GAG binding to TACI is a little controversial. Few studies report that TACI interacts with proteoglycan [32,33,34]. However, some studies claim no binding of TACI to HSPG [35] or find it unlikely [13].

The docking of such long GAGs in a biologically relevant system has been performed for the first time to our knowledge. In this work, we analyzed the data obtained with RS-REMD for APRIL–BCMA/TACI–HP complexes and compared the docking performance and predictive power of this method to the ones of the conventional molecular docking method (Autodock3). Our results contribute to the general knowledge about GAG-specific computational approaches.

## 2. Materials and Methods

### 2.1. Structures

Protein structures. Following X-ray experimental structures from PDB were used in this work: 1XU2 (the crystal structure of APRIL bound to BCMA) and 1XU1 (the crystal structure of APRIL bound to TACI) [36].

GAG structures. HP dp24 and dp48—dp stands for the degree of polymerization—were constructed from building blocks of the sulfated GAG monomeric units’ libraries [37] compatible with AMBER16 package based on the experimental structure of HP (PDB ID: 1HPN). The GLYCAM06 force field [38] and the literature data for the sulfate groups [39] were the sources of GAGs’ charges.

### 2.2. Molecular Docking

Autodock3 was used as a standard docking tool, as it has been previously described to yield the best results for GAG–protein complexes [19,40]. Entire protein was covered using a maximum gridbox size (126 Å × 126 Å × 126 Å) with a 0.853 Å grid step. The size of 300 for the initial population and 10^5^ generations for termination conditions were chosen. One thousand independent runs with the Lamarckian genetic algorithm were used. In total, 9995 × 10^5^ energy evaluations were performed. DBSCAN algorithm [41] was used for clustering. RMSatd metric was used for clustering, which accounts for the equivalence of the atoms of the same atomic type. This metric was reported to be more appropriate for GAG docking than classical RMSD for periodic ligands [20].

RS-REMD (replica exchange with repulsive scaling) [22] was used as an effective docking alternative to Autodock3 [24]. The ff14SBonlysc force field parameters [42] for protein and the GLYCAM06 [38] for GAGs were used, respectively. Every step of the docking simulation was followed, as described in detail in the work of Maszota et al. [24], and all the parameters were used as described there.

### 2.3. Molecular Dynamics

All the MD simulations of the complexes obtained by Autodock3 docking were performed in AMBER16 package [43]. TIP3P truncated octahedron water box with a distance of 8 Å from the solute to the box’s border was used to solvate complexes. Na+ counterions were used to neutralize the charge of the system. Energy minimization was performed preceding the production MD runs. 500 steepest descent cycles and 10^3^ conjugate gradient cycles with 100 kcal/mol/Å^2^ harmonic force restraint on solute atoms were performed. It was followed by 3 × 10^3^ steepest descent cycles and 3 × 10^3^ conjugate gradient cycles without any restraints and continued with heating up the system to 300 K for 10 ps with harmonic force restraints of 100 kcal/mol/Å^2^ on solute atoms. Then, the system was equilibrated at 300 K and 10^5^ Pa in an isothermal, isobaric ensemble for 500 ps. The actual MD runs were carried out using the same isothermal, isobaric ensemble for 100 ns. The timestep of 2 fs, the cut-off of 8 Å for electrostatics were used. Particle mesh Ewald method for treating electrostatics [44] and SHAKE algorithm for all the covalent bonds containing hydrogen atoms [45] were implemented in the MD simulations. Cpptraj module of AMBER was used for the analysis of the trajectories. In particular, native contacts command with default parameters was used for the analysis of the contacts between protein and GAG molecules established in the course of the simulation.

### 2.4. Binding Free Energy Calculations

For the free energy and per residue energy decomposition calculations, the MM/GBSA (molecular mechanics generalized born surface area) model igb = 2 [46] from AMBER16 was used on trajectories obtained from MD simulations. These energy values should be rather understood as the enthalpy of binding rather than strictly defined binding free energy and partially include the entropic contribution of the solvent. It was previously shown that for the MM/PBSA (MM/GBSA) approach, entropy calculations would rather increase the uncertainty of the calculated free energy values, in general [47], and for protein–GAG systems, in particular [48]. LIE analysis with a dielectric constant of 80, performed by CPPTRAJ scripts on the same frames as the MM/GBSA.

## 3. Results and Discussion

### 3.1. Docking Heparin dp24 to APRIL–BCMA and APRIL–TACI Complexes Using Autodock3

The internal limitation of Autodock3 allowed specification of only 32 torsional degrees of freedom for the docked ligand as free to rotate, rendering flexible docking of longer (over dp6 or dp8) GAGs unfeasible. Due to this technical limitation of the software, in the performed docking with Autodock3, the central part of the heparin molecules was kept flexible, while the rest remained rigid. Only central 16 glycosidic linkages, each described by two dihedral angles, were flexible, while other degrees of freedom were not considered in the docking run. For these partially rigid docked molecules, we observed some heparin-binding poses for which the overall oligosaccharide backbone geometry did not look correct. Some of them seemed highly unlikely to occur from an energetic standpoint, considering how spatially close the monosaccharide units with the same charge were placed. After visual analysis of the top 50 docked solutions (in terms of energy scores of Autodock3), we selected three and five ones for APRIL–BCMA and APRIL–TACI, respectively, that looked particularly distorted. (Figures S1 and S2) This suggests that the internal ligand conformations are not properly scored by Autodock3; otherwise, such binding poses might not have been included within the energetically favorable ones. Since it is known that Autodock3 can potentially generate glycosidic linkages with inappropriate geometry [49], we checked if the global distortions of the docked HP chains could be related to the locally distorted geometry of the glycosidic linkages (Figure S3). However, all the glycosidic linkages obtained with Autodock3 were located in the same regions as the glycosidic linkages for the unbound HP molecules from the microsecond-range MD simulations, suggesting that the global distortion of long HP molecules in Autodock3 are independent of the local glycosidic linkage geometry that is correct in the analyzed cases. Therefore, the unexpected overall geometries of long HP ligands are originated from another Autodock3 feature.

**Figure 1:**
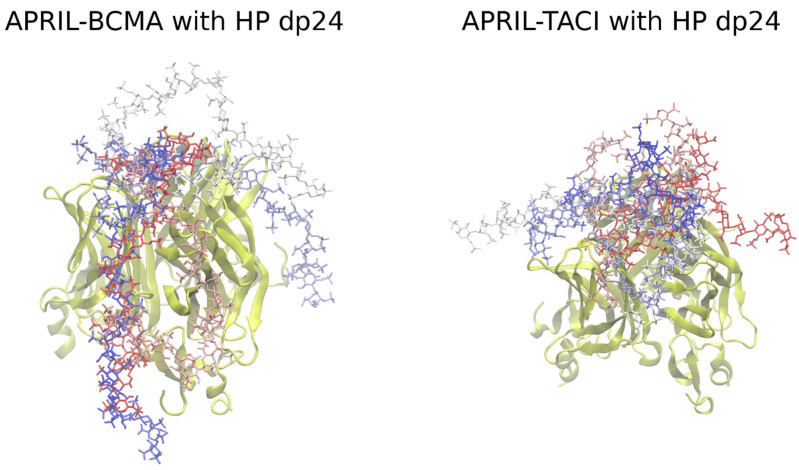
The five most energetically favorable structures of the APRIL–BCMA (**left**) and APRIL–TACI (**right**) complexes with heparin dp24, obtained by docking with Autodock3 (heparin in licorice representation, red-white-blue range of color; APRIL, BCMA, TACI in cartoon representation in yellow).

The best five poses in terms of energy (as scored by Autodock3) were further analyzed with the MD approach for both complexes. (Figure 1) These solutions were highly heterogeneous, though they all were partially located at the GAG binding site on the APRIL molecule, similar to our previous findings obtained in the absence of BMCA/TACI receptors [13]. This suggests that a long GAG would first bind the APRIL molecule independently of the presence of the receptors.

**Figure 2:**
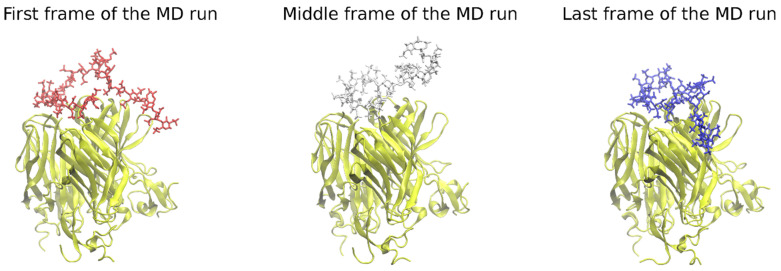
The heparin movement on the protein’s surface in the course of the MD run. Some parts of the HP tend to move substantially during MD runs, searching for an energetically more favorable pose (heparin in licorice representation, red, white, and blue color; APRIL, BCMA, TACI in cartoon representation, yellow color).

The conformations of the heparin over the course of the MD runs starting from the structures obtained by Autodock3 and expressed by RMSD shows from medium to high values (9.1 ± 4.7 Å and 7.3 ± 1.9 Å for APRIL–BCMA and APRIL–TACI, respectively), indicating that these starting conformations did not correspond to the structures favored by the GLYCAM06 force field used in the MD (Table S1). When also taking into account the movement of the GAG on the protein surface, the RMSD values were 15.5 ± 4.7 Å and 11.7 ± 1.8 Å for APRIL–BCMA and APRIL–TACI complexes, respectively. It is worth noting that the part of the HP that seems to be docked properly in the GAG binding region of the APRIL protein remained stable (trapped in a minimum) during the MD runs and, thus, did not significantly contribute to the high RMSD (Figure 2). Therefore, lateral parts of the GAG molecule potentially have been docked wrongly, rendering intra- and intermolecular interactions to be unfavorable and forcing GAG to search for a more energetically convenient pose in the MD simulations. The rigidity of these parts could practically be the reason for such an observation. The evolution of radius of gyration (Rgyr) of the HP dp24 and the whole protein–HP complex, as well as RMSD of the HP molecule (Figures S4 and S5, respectively), show that, except for one of the MD simulations of one BCMA complex, both Rgyr and RMSD converge through the 100 ns of the MD simulation.

Binding free energy analysis using LIE yielded mean energy values of −102.2 ± 17.8 kcal/mol and −125.8 ± 13.4 kcal/mol for the APRIL–BCMA and APRIL–TACI systems, respectively. There is an expected significant difference [13] in the absolute values between the LIE data and the data obtained using MM/GBSA, which yielded more favorable energies: −169.7 ± 34.3–244.9 ± 15.5 kcal/mol APRIL–BCMA and for APRIL–TACI, respectively. These differences are expected to be higher in a case when the system is highly charged since, within the MM/GBSA procedure, minimization is performed for all frames, leading to overestimation of the binding strength of the complex. At the same time, the LIE protocol should be optimized in terms of the dielectric constant for each particular molecular system, which requires experimental data. Energies obtained with MM/GBSA for the first and the last 10 ns were compared for both complexes. For the APRIL–BCMA complex, they were −155.5 ± 22.5 kcal/mol at the start and −194.3 ± 33.9 kcal/mol at the end of the runs, respectively. The data for the APRIL–TACI complex follows the same trend: the corresponding energies were −217.3 ± 28.9 kcal/mol at the start and −255.8 ± 38.0 kcal/mol at the end of the runs, respectively. A decrease in binding energy by such a significant amount suggests that starting conformations were far from being optimal. To sum up, Autodock3, when applied to long GAGs such as HP dp24, yields many artifacts that are partially repaired in the followed MD step. However, even long conventional MD simulations are not able to globally change the conformation of the docked GAG within the practically accessible times.

### 3.2. Docking Heparin dp24 and dp48 to APRIL–BCMA and APRIL–TACI Complexes Using RS-REMD

**Figure 3:**
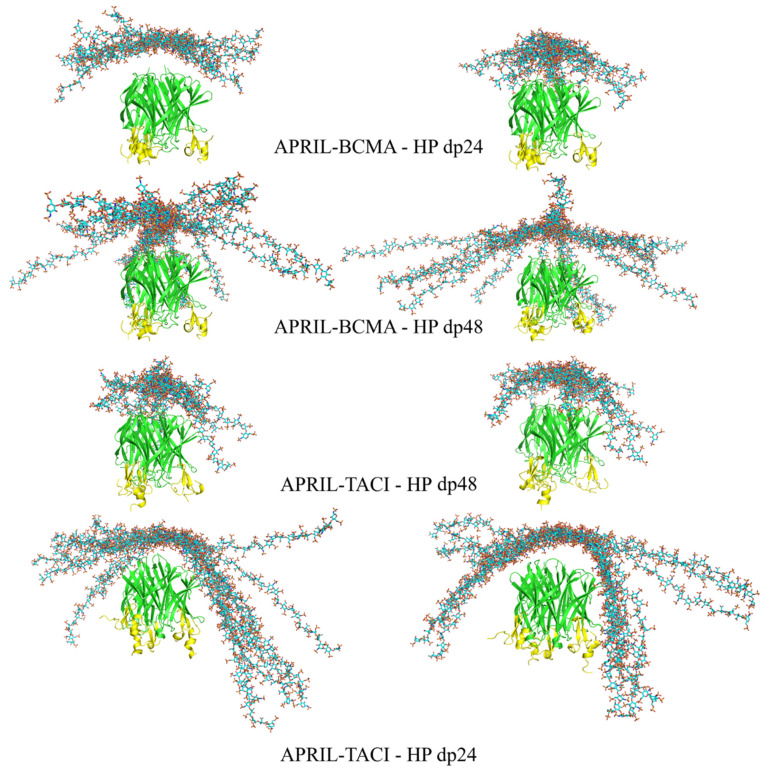
The most energetically favorable (**left**) and manually chosen (**right**) structures of the APRIL–BCMA and APRIL–TACI complexes with HP dp24 and HP dp48 after RS-REMD simulation (HP in licorice representation, cyan color; APRIL (green color) and BCMA/TACI (yellow color) in cartoon representation).

RS-REMD simulations for HP dp24 and dp48 were performed for both APRIL–BCMA and APRIL–TACE complexes, and the results were compared with the ones obtained from Autodock3 described above. Afterward, a refinement procedure was carried out for the docking poses obtained during the RS-REMD simulation, as was described in detail in our previous work [24]. First, MM-GBSA analysis for the whole RS-REMD trajectories was performed, then 10 docking poses with the lowest electrostatic energy were selected for the refinement procedure, as this free energy component proved to perform better for scoring in protein–GAG systems [24]. At the same time, 10 poses were also selected manually based on the visual criteria that the HP lateral parts were directed toward the BCMA/TACI APRIL receptors to determine if such structures could be energetically favorable in comparison to other ones, which potentially could mean that HP molecules favourize the binding between APRIL and its receptors. All starting structures are presented in Figure 3.

There are no significant differences between the structures selected for the improvement procedure based on the electrostatic energy value and those selected manually. In the case of the HP dp24 ligand, the differences between the APRIL protein with BCMA and TACI receptors are neither visible. In both cases, the ligand is docked at the top of the protein trimer with its middle part, while the ends point toward the solution. In contrast, for the HP dp48 ligand, it can be observed that it still docks at the top of the protein for both APRIL–BCMA and APRIL–TACI, but its ends, especially in the case of APRIL–TACI, are directed toward the receptors. This means that RS-REMD predicts the potential binding of the long GAGs not only to APRIL, which is by far the strongest, but also to the APRIL receptors.

**Figure 4:**
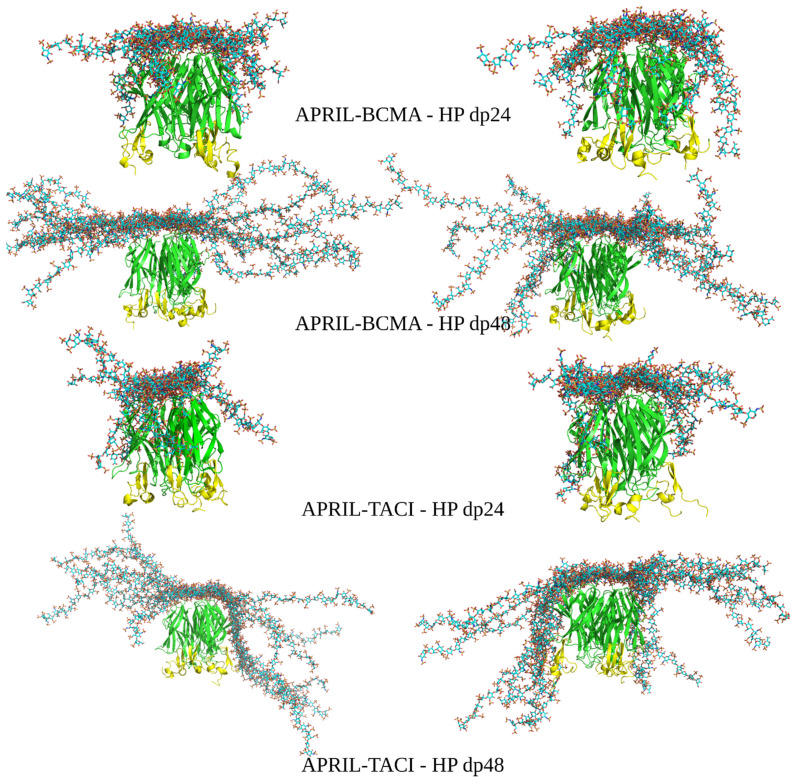
The most energetically favorable (**left**) and manually chosen (**right**) structures of the APRIL–BCMA and APRIL–TACI complexes with HP dp24 and HP dp48 after refinement procedure (HP in licorice representation, cyan color; APRIL (green color) and BCMA/TACI (yellow color) are in cartoon representation.

To refine the contacts between the BCMA/TACI receptors and HP molecules in the explicit solvent, 100 ns and 20 ns of the MD simulations were performed for dp24 and dp48, respectively. The structures of the complexes obtained in the refinement procedure are shown in Figure 4.

It is clearly seen that for all the refined complexes, the distance between the HP ligands and the protein has decreased. The per-frame evolution of HP RMSD and MM/GBSA binding free energy is shown for representative APRIL-BCMA-HP dp24 and APRIL-TACI-HP dp24 refinement MD simulations (Figure S6), where convergence is demonstrated. At the same time, we would like to underline that the work did not aim to achieve proper convergence of the simulations but to obtain an ensemble of docked binding poses using the RS-REMD methodology and to compare its prediction power with the one of Autodock3. The obtained binding poses can be further analyzed by applying longer MD simulations depending on the scientific goal in a particular study, in which an RS-REMD docking scheme is applied as the first step to obtain a starting complex between a protein and a long GAG. For the HP dp24 complexes, no contacts with the BCMA receptor can be observed, while they are visible for the APRIL-TACI-HP dp24 complex. Similarly, the contacts in complexes between APRIL–BCMA/TACI–HP dp48 have been improved. Again, contacts between the HP ligand and the TACI receptor are more frequent than in the case of BCMA.

Contacts evolution for Autodock3-docked and RS-REMD (after the refinement)-docked HP dp24 was compared (Figures S7 and S8). In both procedures, there were more contacts observed in the case of TACI complexes, while the number of contacts does not differ essentially for the Autodock3 and RS-REMD MD simulations. For Autodock3, there are already existing contacts (initial contacts), while due to the nature of the RS-REMD procedure [24], there are barely any initial contacts prior to the refinement.

Furthermore, we analyzed the dependence of the calculated binding energies obtained by Autodock3-based and RS-REMD approaches on the angle defined by the terminal (the first) monosaccharide unit of HP-central atom of the HP chain-the middle of the HP molecule-the terminal (the last) monosaccharide unit of HP (Figure S9). For both BCMA and TACI complexes, Autodock3 results show a less pronounced preference of the binding energies for the lower angle values, meaning that the bending of the GAG around the ARPIL–receptor complex is less favored than in the case of the RS-REMD procedure.

**Figure 5:**
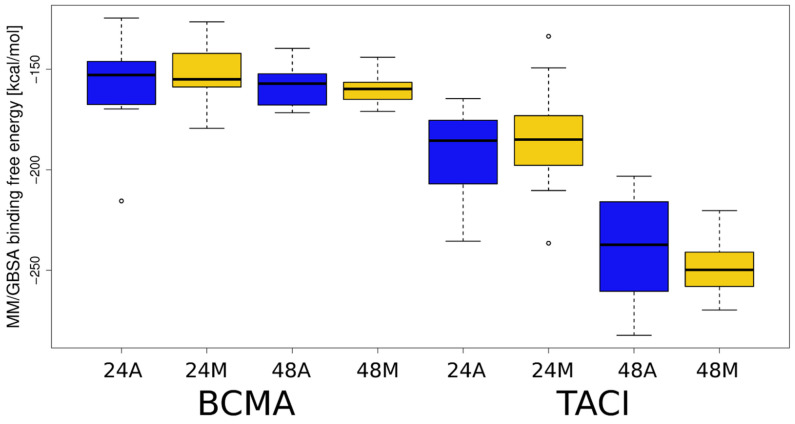
MM/GBSA binding free energies obtained for APRIL–BCMA/TACI–HP systems. Boxplot representation for the values provided in Table S2 is plotted: X-axis, BCMA and TACI correspond to the APRIL complexes with BCMA and TACI, respectively; 24 and 48 correspond to HP dp24 and HP dp48, respectively; A and M correspond to the automatically and manually selected structures, respectively.

To discover which of the analyzed complexes have the most favorable energy, as well as which receptor amino acid residues are responsible for binding HP, MM-GBSA binding and per residue decomposition analysis were performed. The results of the MM-GBSA analysis are shown in Figure 5, Table S2. For all analyzed complexes, the free energy of binding is more favorable for HP dp48 than for HP dp24, suggesting that despite the core binding site on the top of the APRIL–receptor complexes, the elongation of a GAG essentially strengthens the binding. Significant differences in ∆G value are noticeable for APRIL-BCMA-HP and APRIL-TACI-HP complexes. The mean free energy of binding for the APRIL–BCMA complexes is about 25% less favorable than for the APRIL-TACI-HP complexes. We observed the same trend when GAGs were docked with Autodock3. It is worth noting that for the APRIL-BCMA-HP complexes, there are noticeable differences between the energies of the structures selected on the basis of the RS-REMD electrostatic energy value and those selected manually. However, in the case of APRIL-TACI-HP, the differences are practically imperceptible.

Next, per residue decomposition was performed allowingto identify the most energetically favorable BCMA/TACI amino acid residues involved in the formation of the complexes. The amino acid residues with the decomposed binding free energies below −1.5 kcal/mol are listed in Table S3.

The number of the BCMA/TACI amino acid residues involved in complex formation with HP increased with the increasing ligand length. This is due to the possibility of contacts between HP dp48 and several receptor monomers simultaneously, while HP dp24 is too short to be able to interact with several receptors at once. Free energy values decrease with ligand length for both APRIL–BCMA and APRIL–TACI proteins. Importantly, the free energy values take lower values for APRIL–TACI than for APRIL–BCMA: the lowest and the average values for APRIL-BCMA-HP dp24, APRIL-TACI-HP dp24, APRIL-BCMA-HP dp48, and APRIL-TACI-HP dp48 are −2.2 kcal/mol and −1.9 ± 0.2 kcal/mol, −3.8 kcal/mol and −2.1 ± 0.6 kcal/mol, −3.1 kcal/mol and −2.9 ± 0.2 kcal/mol, and −11.8 kcal/mol and −4.3 ± 2.0 kcal/mol, respectively. All these results suggest that in the case when a long GAG molecule is indeed able to bind simultaneously to both APRIL and its receptors, this would strengthen the interactions in an APRIL–receptor complex. Such an effect would be more pronounced in the case of TACI binding to APRIL. This is in agreement with our previous simulations indicating that TACI is more prone to bind GAGs than BCMA [13].

## 4. Conclusions

Long (dp24 and dp48) heparin molecules were successfully docked to the binding site of the APRIL–BCMA and APRIL–TACI complexes. In the case of the APRIL–TACI complex with heparin, we observed stronger binding than in the APRIL–BCMA complex. In all cases, the GAG is first bound to the APRIL GAG binding site, while secondary interactions are established with the receptors. It is puzzling, however, how TACI, especially remotely located from a GAG in the case of the HP dp24, could substantially impact GAG binding by the APRIL protein. Probably this is due to the long-range electrostatics effect occurring in this highly charged system. At the same time, multiple studies find no or, rather, unlikely binding of GAGs to TACI [13,35]. We conclude that the TACI–GAG interaction could be amplified by TACI’s binding to APRIL, while this interaction was not detected in the absence of APRIL. In this study, RS-REMD showed clear superiority over Autodock3 in the case of docking quality for such long molecules. First of all, it is almost unfeasible to dock dp48 GAGs properly with Autodock3, while docking dp24 experiences essential challenges. However, we have to admit that despite some geometrically inappropriate docking poses in the case of Autodock3 when applied for HP dp24, most of the docking structures were not distorted. Nonetheless, they showed higher instability of the HP binding poses in the course of the MD simulations than in the case of RS-REMD, suggesting worse sampling during the docking by Autodock3. Considering the fact that RS-REMD yielded better results without the need for more computational resources for a representative protein–GAG dataset [24], we strongly believe that this method is far more superior in the case of docking long GAG molecules.

## Data Availability

Not applicable.

## Supplementary Materials

The following are available online at https://www.mdpi.com/article/10.3390/biom11091349/s1, Table S1: RMSD values from the performed MD runs. RMS-GAG means RMSD of the GAG molecule in respect to its starting pose, when RMS-GAG-PROTEIN additionally include GAG position in respect to protein, Table S2: MM-GBSA analysis for the APRIL-BCMA/TACI-HP MD simulations, Table S3: MM-GBSA per residue decomposition analysis for the APRIL-BCMA/TACI-HP MD simulations. The residues belonging to BMCA/TACI molecules are only listed. The letter after the residue corresponds to the monomeric unit within the trimer, Figure S1: Five docked posed selected out of top 50 (in terms of energy evaluation with Autodock3) that were distorted in terms of the global heparin geometry for APRIL-BCMA complex (heparin in licorice representation, cyan colour; APRIL and BCMA in cartoon representation, yellow colour), Figure S2: Five docked posed selected out of top 50 (in terms of energy evaluation with Autodock3) that were distorted in terms of the global heparin geometry for APRIL-TACI complex (heparin in licorice representation, cyan colour; APRIL and TACI in cartoon representation, yellow colour), Figure S3: Glycosidic linkage heatmaps for HP φ and ψ dihedral angles defined as O5n+1-C1n+1-O4n-C4n and C1n+1-O4n-C4n-C4n where n stands for sequential number of a sugar monomeric unit, the data are obtained from 5 μs MD simulation for the unbound HP dp10. The data for glycosidic linkages of each type (GlcNS(6S)-IdoA(2S) in A and GlcNS(6S)-IdoA(2S) in B) are averaged. The data for glycosidic linkages for one of the distorted poses of HP dp24 docked to APRIL/BCMA obtained with Autodock3 are in magenta points, Figure S4: APRIL-BCMA-HP dp24 (left panel) and APRIL-TACI-HP dp24 (right panel) per frame Rgyr analysis, Figure S5: APRIL-BCMA-HP dp24 (left panel) and APRIL-TACI-HP dp24 (right panel) per frame RMSD analysis, Figure S6: Per frame evolution of HP MM/GBSA binding free energy and RMSD for two representative APRIL-BCMA-HP dp24 (left panel) and APRIL-TACI-HP dp24 (right panel) RS-REMD refinement MD simulations corresponding to the lowest free energy values, Figure S7: AD3-predicted APRIL-BCMA-HP dp24 (left panel) and APRIL-TACI-HP dp24 (right panel) contact number analysis, Figure S8: RS-REMD-predicted APRIL-BCMA-HP dp24 (left panel) and APRIL-TACI-HP dp24 (right panel) contact number analysis, Figure S9: MM/GBSA free binding energy dependence on the angle defined by the terminal (the first) monosaccharide unit of HP-central atom of the HP chain-the terminal (the last) monosaccharide unit of HP obtained by AD3 and RS-REMD approaches for APRIL-BCMA-HP dp24 (left panel) and APRIL-TACI-HP dp24 (right panel) complexes.
