# Supplementary material for: Advanced Molecular Dynamics Approaches to Model a Tertiary Complex APRIL/TACI with Long Glycosaminoglycans
Source: Biomolecules. 2021 Sep 12;11(9):1349. doi: 10.3390/biom11091349 (PMC8465899; doi:10.3390/biom11091349)
Supplement: Supplementary file 1 [file biomolecules-11-01349-s001.zip › biomolecules-1313498-supplementary.pdf]

Supplementary Information

Table S1. RMSD values from the performed MD runs. RMS-GAG means RMSD of the GAG molecule in respect to its starting pose, when RMS-GAG-PROTEIN additionally include GAG position in respect to protein.

**REMD-RS**					
**Complex variant**		**Lowest REMD-RS Electrostatic Energies**		**Manually Chosen**	
		**rms_GAG**	**rms_GAG_protein**	**rms_GAG**	**rms_GAG_protein**
APRIL-TACI-HP dp24	1	6.1	9.2	5.2	10.2
	2	4.8	9.9	6.1	12.1
	3	6	9.8	5.6	10.2
	4	7.7	12.2	6.6	9.2
	5	5.6	9.9	5.6	10.3
	6	6.5	10.6	6.3	10.9
	7	5.8	10.2	6.4	12.7
	8	6.4	11.5	6.1	11.0
	9	7	11.0	7.3	10.3
	10	4.8	11.0	5.3	11.2
	average	6.1	10.5	6.0	10.8
APRIL-BCMA-HP dp24	1	7.9	11.9	7.5	12.0
	2	4.9	14.6	4.4	11.7
	3	6.3	11.0	6.0	11.0
	4	5.3	8.9	7.8	14.2
	5	5.7	8.2	6.1	16.9
	6	4.7	10.9	5.4	10.1
	7	4.7	12.2	5.7	11.8
	8	5	8.4	7.7	12.1
	9	4.6	11.0	6.3	10.2
	10	4.5	8.1	5.7	12.0
	average	5.4	10.5	6.3	12.2
Autodock3					
Complex variant		rms_GAG	rms_GAG_protein		

APRIL-TACI-HP dp24	1	9.6	13.5	
	2	7.9	10.3	
	3	6.6	11.7	
	4	8.1	12.0	
	5	4.5	8.8	
	average	7.3	11.3	
APRIL-BCMA-HP dp24	1	8.9	10.8	
	2	8.1	15.5	
	3	8.8	21.5	
	4	14.2	18.5	
	5	5.5	11.1	
average		9.1	15.5	

Table S2. MM-GBSA analysis for the APRIL-BCMA/TACI-HP MD simulations.

complex variant	lowest REMD-RS electrostatic energies		manually chosen	
	ΔG [kcal/mol]	average ΔG [kcal/mol]	ΔG [kcal/mol]	average ΔG [kcal/mol]
APRIL-BCMA-HP dp24	−215.5	−157.0	−152.3	−152.1
	−146.1		−158.2	
	−154.8		−138.4	
	−124.5		−144.1	
	−150.9		−142.1	
	−137.6		−126.4	
	−148.4		−163.4	
	−154.8		−179.4	
	−167.5		−158.8	
	−169.7		−157.7	
APRIL-BCMA-HP dp48	−162.2	−158.0	−163.9	−159.4
	−155.8		−146.8	
	−158.5		−144.0	
	−139.6		−156.5	
	−171.6		−170.5	
	−155.5		−156.7	
	−168.1		−165.0	
	−167.8		−162.9	
	−152.3		−171.0	
	−148.3		−156.6	
APRIL-TACI-HP dp24	−178.2	−192.5	−173.1	−184.1
	−164.6		−133.6	
	−185.5		−210.3	
	−194.5		−183.6	
	−223.5		−236.5	
	−207.0		−186.3	
	−175.1		−180.4	
	−235.5		−189.9	
	−185.5		−149.3	
	−175.4		−197.8	
APRIL-TACI-HP dp48	−215.9	−240.8	−241.0	−248.4
	−224.3		−269.7	
	−203.2		−238.3	
	−222.1		−247.7	
	−282.2		−251.9	
	−252.4		−241.8	
	−260.4		−254.1	
	−215.6		−220.3	
	−250.3		−258.0	
	−281.8		−260.8	

Table S3. MM-GBSA per residue decomposition analysis for the APRIL-BCMA/TACI-HP MD simulations. The residues belonging to BMCA/TACI molecules are only listed. The letter after the residue corresponds to the monomeric unit within the trimer.

Complex Variant	Lowest REMD-RS Electrostatic Energies			Manually Chosen		
	Amino Acid Residue		ΔG [kcal/mol]	Amino Acid Residue		ΔG [kcal/mol]
APRIL-BCMA-HP dp24	CYX A	8	−1.9	CYX A	8	−2.0
	ARG A	27	−2.0	ARG A	27	−2.1
	ARG A	39	−1.7	ARG A	39	−1.8
	GLY B	2	−2.2	GLY B	2	−2.1
	ARG B	27	−2.1	ARG B	27	−2.1
	ARG B	39	−1.7	ARG B	39	−1.7
	CYX C	8	−2.0	CYX C	8	−1.8
	ARG C	27	−2.0	ARG C	27	−2.0
	ARG C	39	−1.7	ARG C	39	−1.8
APRIL-BCMA-HP dp48	CYX A	8	−3.0	CYX A	8	−3.0
	ARG A	27	−3.0	ARG A	27	−3.1
	ARG A	39	−2.7	ARG A	39	−2.8
	GLY B	2	−3.0	GLY B	2	−3.0
	ARG B	27	−3.0	ARG B	27	−3.0
	ARG B	39	−2.6	ARG B	39	−2.7
	CYX C	8	−2.8	CYX C	8	−3.0
	ARG C	27	−2.9	ARG C	27	−3.0
	ARG C	39	−2.6	ARG C	39	−2.7
APRIL-TACI-HP dp24	SER A	86	−2.3	SER A	86	−1.8
	ARG A	72	−1.7	ARG A	72	−1.6
	LYS A	73	−1.8	LYS A	73	−1.7
	LYS A	77	−1.9	LYS A	77	−1.8
	ARG A	84	−3.1	ARG A	84	−2.6
	LYS A	98	−1.9	LYS A	98	−1.9
	CYX B	71	−2.6	CYX B	71	−2.2
	ARG B	72	−3.3	ARG B	72	−3.8
	LYS B	73	−2.3	LYS B	73	−2.0
	LYS B	77	−1.9	LYS B	77	−1.9
	ARG B	84	−3.6	ARG B	84	−2.7
	LYS B	98	−2.0	LYS B	98	−2.0
	CYX C	71	−1.6	CYX C	71	−1.9
	ARG C	72	−1.6	ARG C	72	−1.9
	LYS C	73	−1.6	LYS C	73	−1.9
	LYS C	77	−1.7	LYS C	77	−1.8
	ARG C	84	−2.3	ARG C	84	−3.0
	LYS C	98	−1.9	LYS C	98	−2.0
	LYS C	107	−1.5	LYS C	107	−1.6
APRIL-TACI-HP dp48	SER A	86	−2.9	SER A	86	−3.7

	ARG A	72	−2.8	ARG A	72	−4.8
	LYS A	73	−3.0	LYS A	73	−3.9
	LYS A	77	−3.0	LYS A	77	−3.5
	ARG A	84	−3.6	ARG A	84	−4.7
	LYS A	98	−3.5	LYS A	98	−3.8
	CYX B	71	−2.8	CYX B	71	−3.2
	ARG B	72	−2.7	ARG B	72	−3.1
	LYS B	73	−2.8	LYS B	73	−3.2
	LYS B	77	−2.9	LYS B	77	−3.3
	ARG B	84	−3.5	ARG B	84	−3.9
	LYS B	98	−3.1	LYS B	98	−3.4
	CYX C	71	−5.7	CYX C	71	−4.6
	ARG C	72	−8.9	ARG C	72	−11.8
	LYS C	73	−6.1	LYS C	73	−8.5
	LYS C	77	−3.9	LYS C	77	−4.5
	ARG C	84	−7.1	ARG C	84	−6.5
	LYS C	98	−3.7	LYS C	98	−3.9
	LYS C	107	−3.7	LYS C	107	−3.9

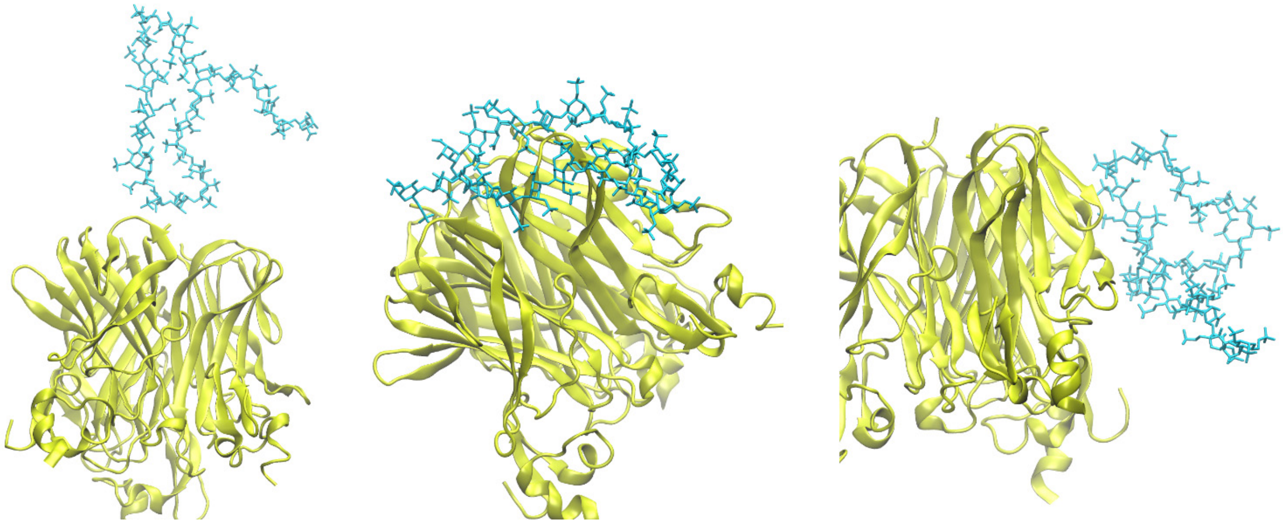

Figure S1. Five docked posed selected out of top 50 (in terms of energy evaluation with Autodock3) that were distorted in terms of the global heparin geometry for APRIL-BCMA complex (heparin in licorice representation, cyan colour; APRIL and BCMA in cartoon representation, yellow colour).

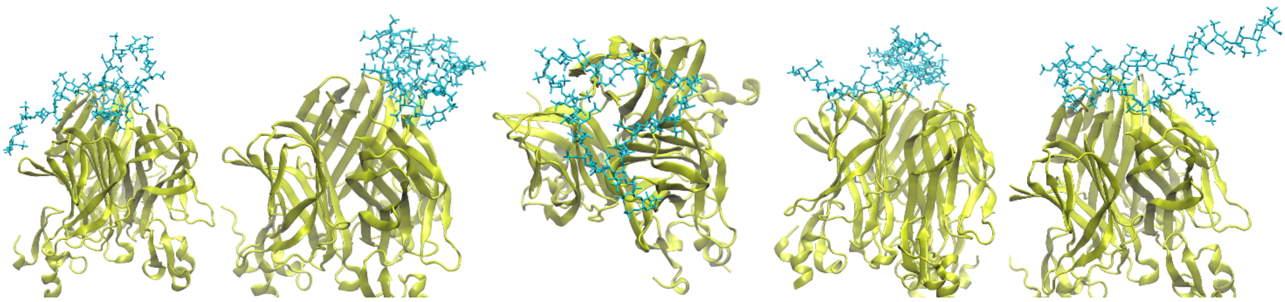

Figure S2. Five docked posed selected out of top 50 (in terms of energy evaluation with Autodock3) that were distorted in terms of the global heparin geometry for APRIL-TACI complex (heparin in licorice representation, cyan colour; APRIL and TACI in cartoon representation, yellow colour).

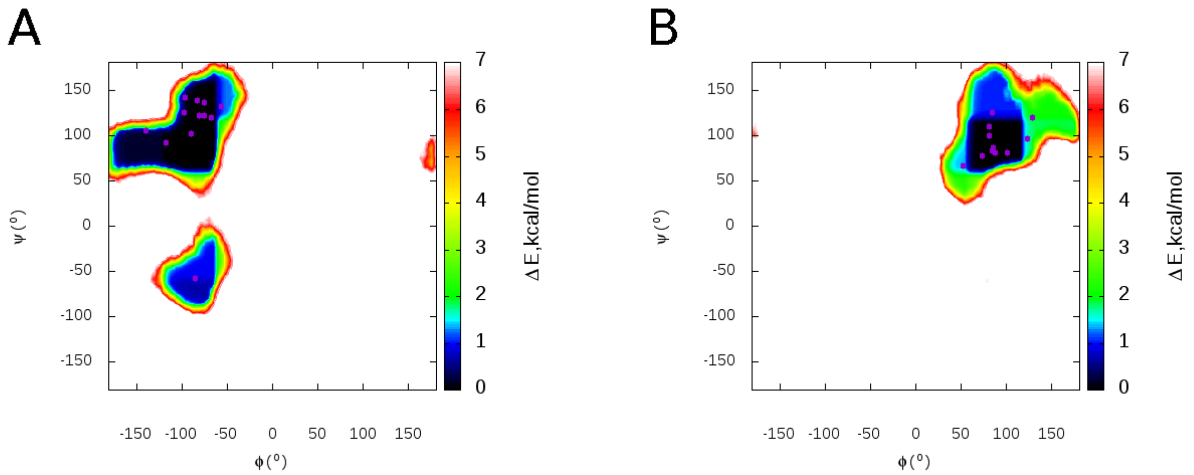

Figure S3. Glycosidic linkage heatmaps for HP φ and ψ dihedral angles defined as $O5_{n+1}$-$C1_{n+1}$-$O4_n$-$C4_n$ and $C1_{n+1}$-$O4_n$-$C4_n$-$C4_n$ where *n* stands for sequential number of a sugar monomeric unit, the data are obtained from 5 μs MD simulation for the unbound HP dp10. The data for glycosidic linkages of each type (GlcNS(6S)-IdoA(2S) in A and GlcNS(6S)-IdoA(2S) in B) are averaged. The data for glycosidic linkages for one of the distorted poses of HP dp24 docked to APRIL/BCMA obtained with Autodock3 are in magenta points.

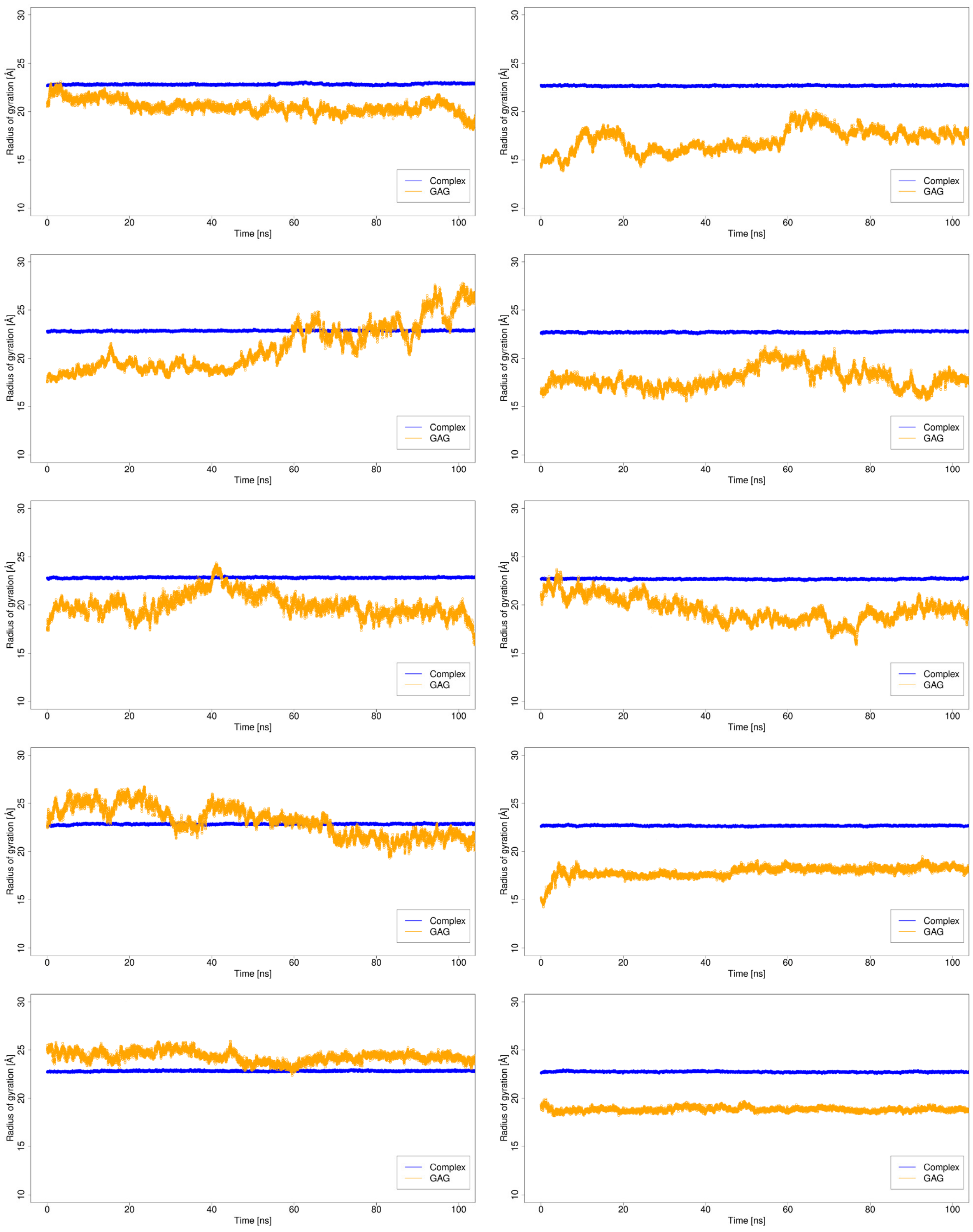

Figure S4. APRIL-BCMA-HP dp24 (left panel) and APRIL-TACI-HP dp24 (right panel) per frame Rgyr analysis.

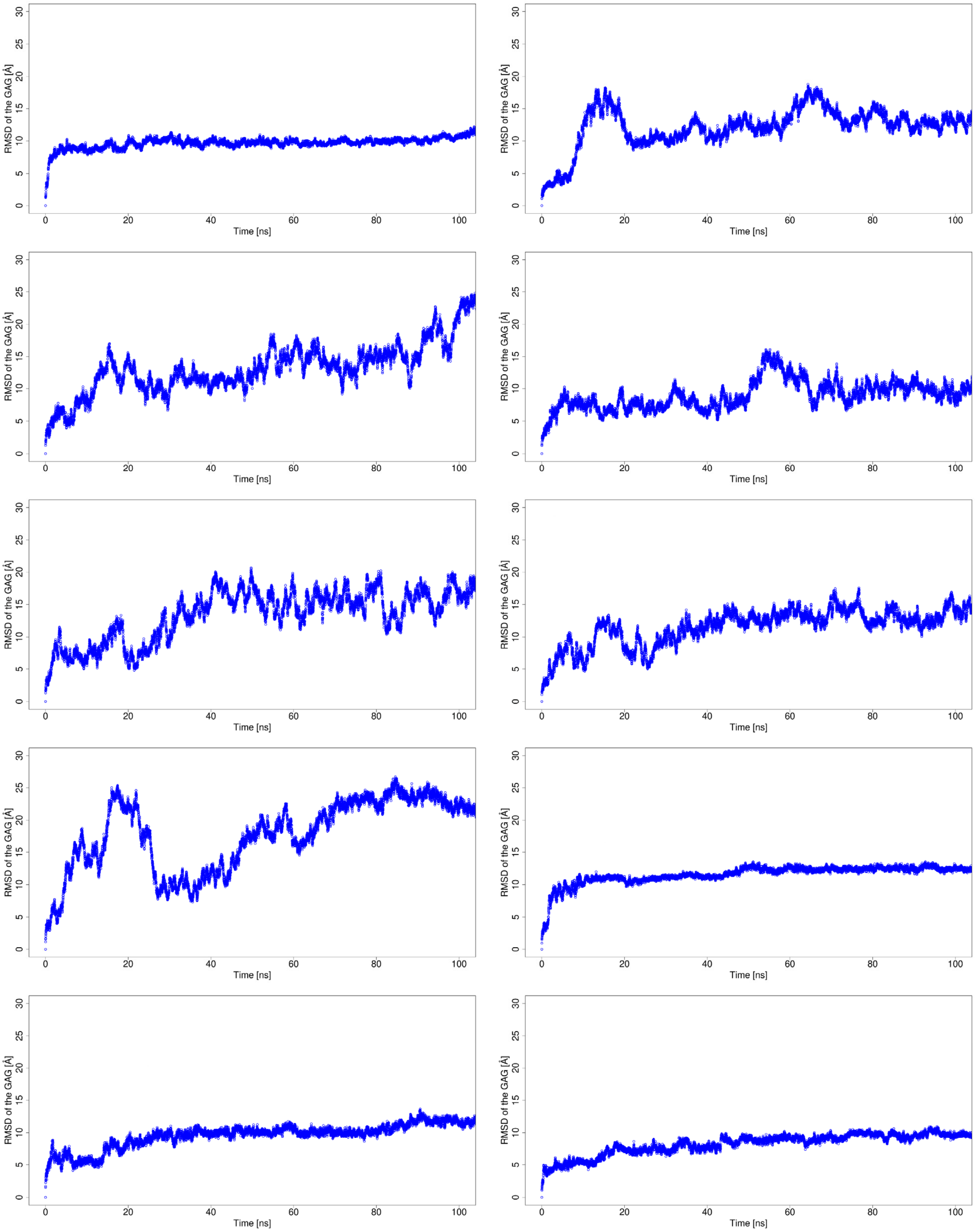

Figure S5. APRIL-BCMA-HP dp24 (left panel) and APRIL-TACI-HP dp24 (right panel) per frame RMSD analysis.

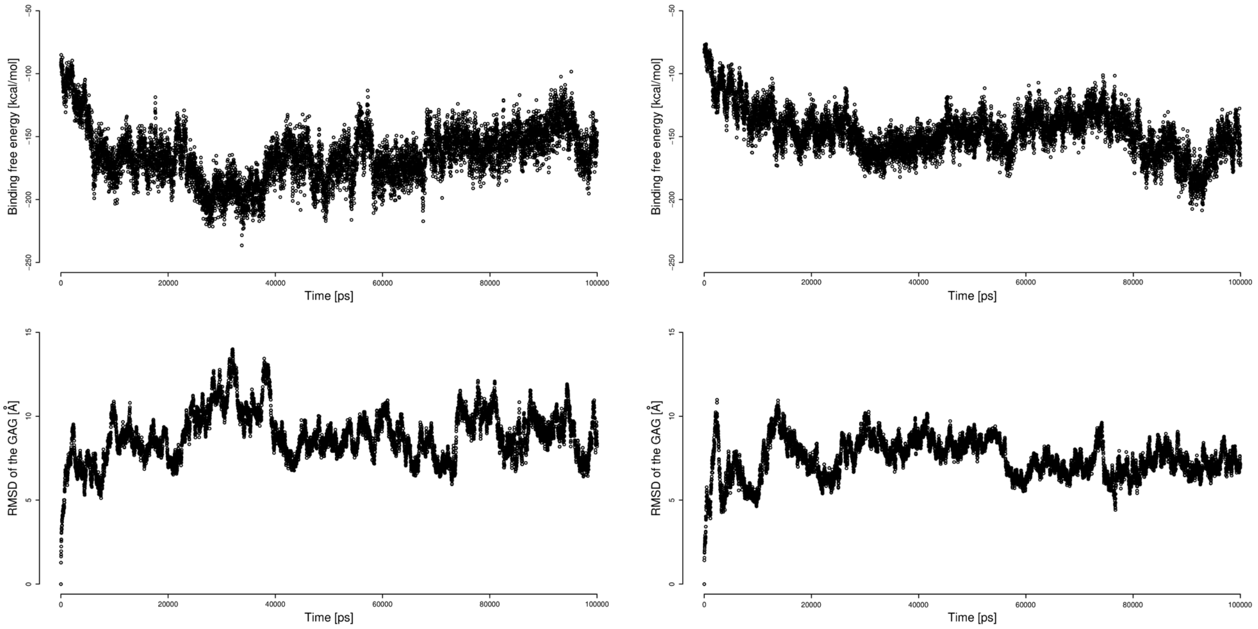

Figure S6. Per frame evolution of HP MM/GBSA binding free energy and RMSD for two representative APRIL-BCMA-HP dp24 (left panel) and APRIL-TACI-HP dp24 (right panel) RS-REMD refinement MD simulations corresponding to the lowest free energy values.

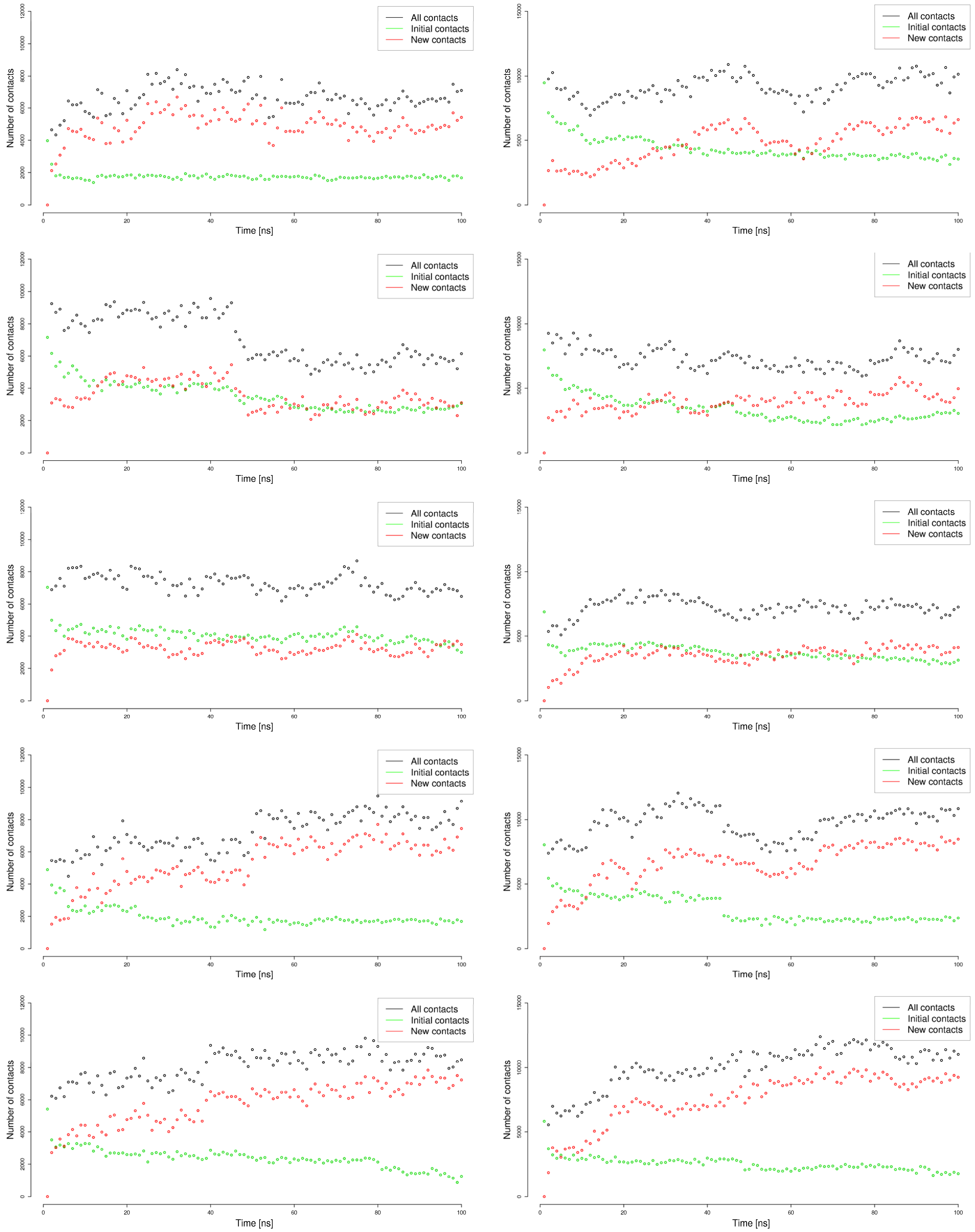

Figure S7. AD3-predicted APRIL-BCMA-HP dp24 (left panel) and APRIL-TACI-HP dp24 (right panel) contact number analysis.

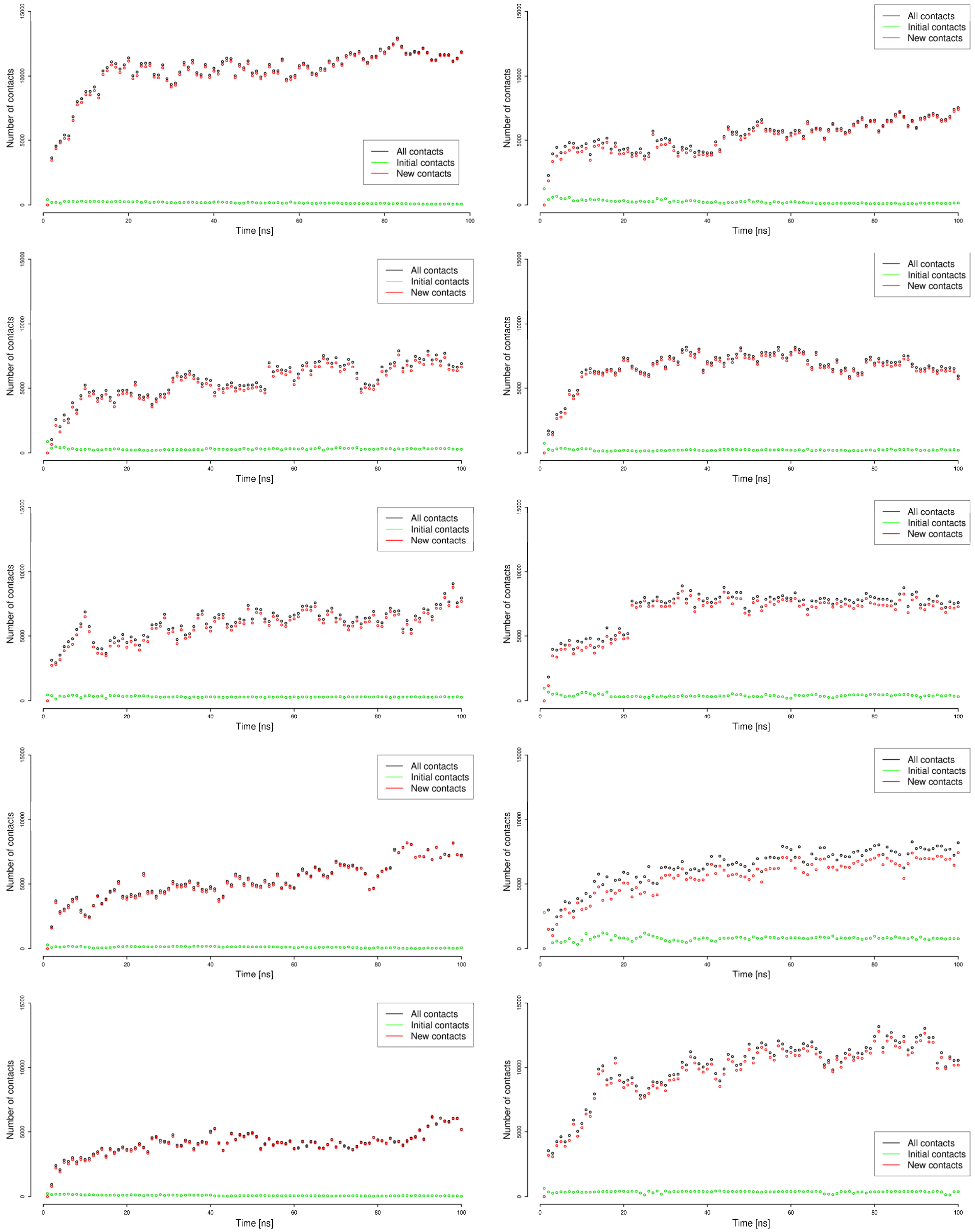

Figure S8. RS-REMD-predicted APRIL-BCMA-HP dp24 (left panel) and APRIL-TACI-HP dp24 (right panel) contact number analysis.

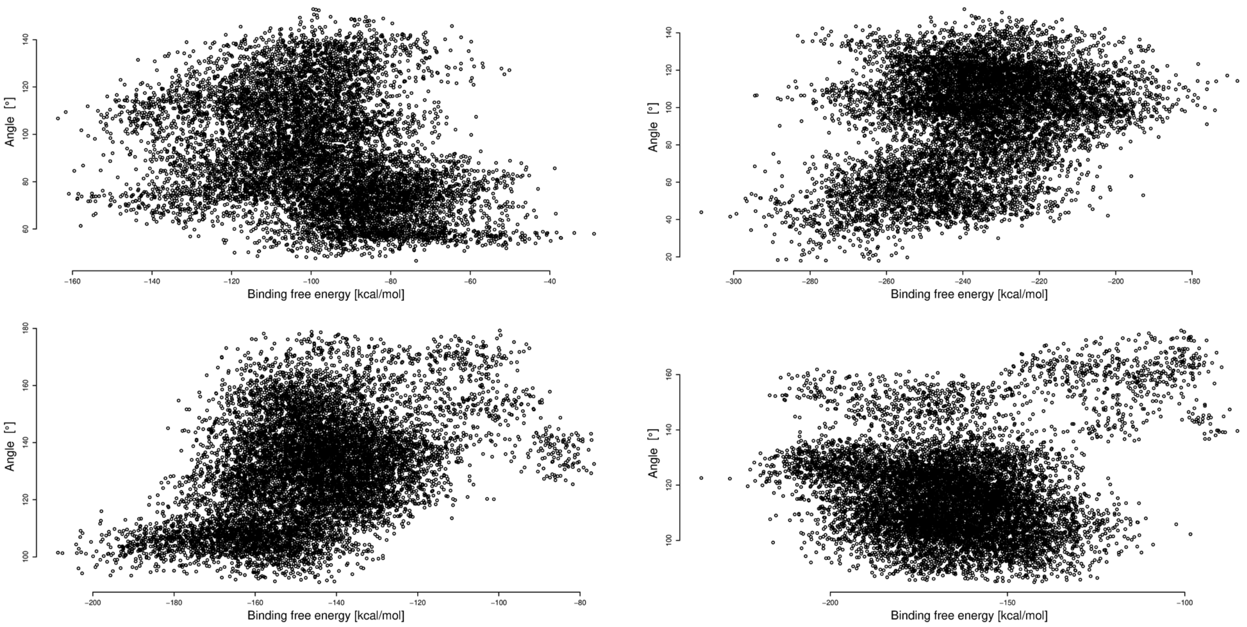

Figure S9. MM/GBSA free binding energy dependence on the angle defined by the terminal (the first) monosaccharide unit of HP-central atom of the HP chain-the terminal (the last) monosaccharide unit of HP obtained by AD3 and RS-REMD approaches for APRIL-BCMA-HP dp24 (left panel) and APRIL-TACI-HP dp24 (right panel) complexes.
